# Motor cortex stimulation in chronic neuropathic orofacial pain syndromes: a systematic review and meta-analysis

**DOI:** 10.1038/s41598-020-64177-z

**Published:** 2020-04-28

**Authors:** Dylan Henssen, Erkan Kurt, Anne-Marie Van Cappellen van Walsum, Tamas Kozicz, Robert van Dongen, Ronald Bartels

**Affiliations:** 1 Department of Radiology, Nuclear Medicine and Anatomy, Radboud university medical center, Nijmegen, The Netherlands; 2 Department of Neurosurgery, Radboud university medical center, Nijmegen, The Netherlands; 3 Unit of Functional Neurosurgery, Radboud university medical center, Nijmegen, The Netherlands; 4 Department of Anesthesiology, Pain and Palliative Care, Radboud university medical center, Nijmegen, The Netherlands

**Keywords:** Neurology, Neurological disorders

## Abstract

Invasive motor Cortex Stimulation (iMCS) was introduced in the 1990’s for the treatment of chronic neuropathic orofacial pain (CNOP), although its effectiveness remains doubtful. However, CNOP is known to be a heterogeneous group of orofacial pain disorders, which can lead to different responses to iMCS. Therefore, this paper investigated (1) whether the effectiveness of iMCS is significantly different among different CNOP disorders and (2) whether other confounding factors can be impacting iMCS results in CNOP. A systematic review and meta-analysis using a linear mixed-model was performed. Twenty-three papers were included, totaling 140 CNOP patients. Heterogeneity of the studies showed to be 55.8%. A visual analogue scale (VAS) measured median pain relief of 66.5% (ranging from 0–100%) was found. Linear mixed-model analysis showed that patients suffering from trigeminal neuralgia responded significantly more favorable to iMCS than patients suffering from dysfunctional pain syndromes (p = 0.030). Also, patients suffering from CNOP caused by (supra)nuclear lesions responded marginally significantly better to iMCS than patients suffering from CNOP due to trigeminal nerve lesions (p = 0.049). No other confounding factors were elucidated. This meta-analysis showed that patients suffering from trigeminal neuralgia and patients suffering from (supra)nuclear lesions causing CNOP responded significantly more favorable than others on iMCS. No other confounding factors were found relevant.

## Introduction

In the early 1990’s, Tsubokawa and his colleagues searched for a new therapy to treat intractable neuropathic pain as other forms of therapies, including continuous deep brain stimulation of thalamic nuclei, only provided satisfactory pain relief in approximately 30% of the cases. For that reason, they started to empirically stimulate various brain regions in animal models for intractable neuropathic pain. During their experiments, they recognized the primary motor cortex as a target that could provide excellent pain relief. Within their experiments, Tsubokawa transected the spinothalamic tract in cats, which led to thalamic hyperactivity and pain-related behavior. By stimulating the primary motor cortex in these cats, it was found that both the hyperactivity was inhibited and the pain-related behavior diminished, indicating an analgesic effect of iMCS in the treated cats. These experimental findings were in line with their subsequent clinical investigations in individuals suffering from intractable neuropathic pain as a consequence of thalamic syndrome^1–5^. Since then, iMCS has been carried out in approximately 700 cases world-wide, yielding highly variable outcomes^1,6–40^. Nevertheless, iMCS has become a last resort neurosurgical therapy for different intractable neuropathic pain syndromes^3–5,14,17,21,26,35,41–44^. In 1993, Meyerson *et al*. reported for the first time on the use of iMCS in chronic neuropathic orofacial pain (CNOP) as a last resort treatment. They reported that, in a case series of 10 individuals suffering from CNOP, iMCS seemed to be a promising treatment^45^. Over the years, various papers have been published discussing both favorable^10,24,46^ and less favorable^9,47^ outcomes of iMCS in treating CNOP (for a narrative review, please see^48^). Due to the mixed results of iMCS, evidence-based inclusion and exclusion criteria for iMCS are lacking^49^, which creates a heterogeneous group of patients.

In order to help establishing inclusion and exclusion criteria, a recent publication from our group aimed to predict iMCS outcome by using artificial intelligence. This paper showed that various predictive variables existed in iMCS for neuropathic pain, including the sex of the patient and the location of the lesion within the nervous system^50^. In addition, a neuroanatomical paper from our group showed that a more extensive trigeminothalamic network is present in the brainstem of humans and could play an important role in pain processing^51,52^. Based on these two papers, we hypothesized that CNOP caused by lesions within the root of the trigeminal nerve and/or within the brain(stem) will respond more favorable to iMCS than lesions of the trigeminal branches. However, in line with our previous paper, other confounding factors are believed to play an important role in the effectivity of iMCS in CNOP. This meta-analysis therefore assessed whether the effectiveness of iMCS is significantly impacted by localization of a lesion within the somatosensory system (i.e., different CNOP disorders respond differently to iMCS). In addition, we assessed whether other confounding factors impacted the outcomes of iMCS in treating CNOP.

## Materials and methods

### Search strategy

A systematical literature search was carried out in various databases (i.e., Pubmed, MEDLINE, Embase, The Cochrane Library and Google Scholar) until October 2018 adhering to PRISMA and MOOSE guidelines^53,54^. An independent, experienced librarian helped in conducting the searches. The search string included: “Electric Stimulation Therapy”; “Facial pain”; “Face pain”; “Headache and facial pain”; “Motor Cortex”; “Motor Cortex Stimulation”; “Neuralgia”; “Neuropathic”; “Pain”; “Pain Management”; “Precentral Cortex “; “Thalamus”; “Trigeminal nerve diseases”; and/or “Trigeminal neuralgia”. Medical Subject Headings (MeSH-) terms were used to enrich the results. Furthermore, the reference list of retrieved articles was cross-referenced to enrich the database. In addition, authors were contacted when papers or specific data were unavailable for the researchers.

**Figure 1:**
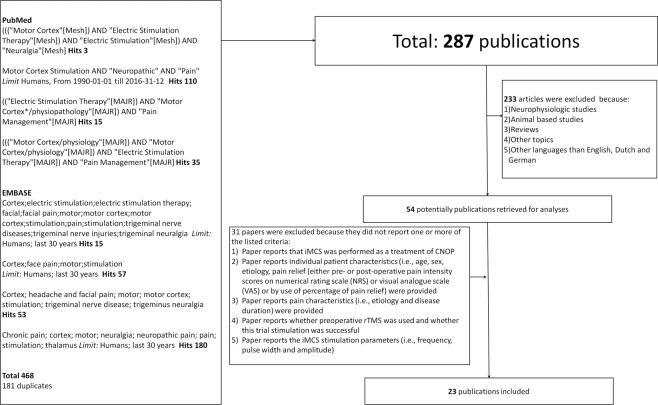
Flow-diagram of the study-selection process. CNOP: Chronic neuropathic orofacial pain.

Original studies (i.e., case-reports, case-series, RCTs) were included when: 1) iMCS was performed as a treatment of CNOP; 2) individual patient characteristics were provided (i.e., age, sex, etiology, pain relief (either pre- or post-operative pain intensity scores on visual analogue scale (VAS)/numerical rating scale (NRS) or by use of percentage of pain relief); and 3) pain characteristics (i.e., etiology and disease duration) were provided. Other papers (e.g., systematic reviews, narrative reviews, technical notes, animal-based studies) were excluded. Furthermore, only papers written in English, Dutch or German were included. Based on these criteria, each article was reviewed for full-text analysis by two researchers independently (D.H. and R.B.). Incongruently assessed papers were reviewed by a third researcher (E.K.), upon which the final decision was made. The selection-process is depicted in Fig. 1 as a flow-diagram.

### Data extraction

Extracted individual patient data included: (1) age, (2) sex, (3) etiology of CNOP, (4) duration of pain, (5) method of iMCS, (6) pain relief post-operatively using VAS or NRS scores or percentages of pain relief, (7) duration of trial phase, (8) complications of iMCS surgery and (9) duration of follow-up of each patient.

Pain relief was categorized by use of a literature-based three-dimensional scale^20^. Level 1, a good pain relief, was defined as a VAS reduction of 70–100%. Level 2 was regarded as satisfactory pain relief and comprised a pain relief lying between 40–69%. Level 3, which indicated a reduction of pain by less than 40%, was defined as a failure. A relevant pain relief was defined as an analgesic effect >40%^20,55^. Patients with less than 40% pain relief were regarded as non-responders^49^. All other values were adapted to nominal numeric input as well. To correct for missing data, the authors of the included papers were contacted in an attempt to obtain missing values. Five feed-forward imputations were carried out by pooling the individual patient data in order to impute missing values.

To assess whether localization of the lesion within the somatosensory system influenced the outcome of iMCS, an anatomical-functional subdivision into 4 categories was made. These groups were:

1. absence of an intrinsic trigeminal nerve lesion (no sensory deficit, no nerve lesion on imaging) and cause of trigeminal nerve hyperexcitability (affecting either pre- or post-ganglion trigeminal fibers up to the nuclei) (e.g., trigeminal neuralgia);
2. trigeminal nerve lesion (affecting either pre- or post-ganglion trigeminal fibers up to the nuclei), resulting in partial or total sensory deficit (e.g., trigeminal neuropathic pain);
3. (supra)nuclear lesion affecting the central sensory pathways between the cortex and the trigeminal nuclei (e.g., post-stroke pain);
4. dysfunctional pain syndrome with central sensitization in the absence of neurological lesion or disproportionate to a possible peripheral lesion (atypical facial pain).

### Statistical assessment

Statistical analyses were carried out by use of IBM SPSS Statistics version 25 (*IBM Corp. Released 2017. IBM SPSS Statistics for Windows, Version 25.0. Armonk, NY: IBM Corp.)*. Three experts in biomedical statistics (TH, JH and MB) were independently involved in choosing and conducting the analyses. A linear mixed model analysis was fitted in order to include a study random effect that accounts for within-study dependency across the included patients. Furthermore, the influence of discordances between the studies and the in-between study variation was corrected by use of the applied linear mixed model. To identify outcome predictive factors and/or confounders regarding the analgesic effect of iMCS in treating CNOP, a linear mixed model analysis was applied to analyze the differences in mean pain relief in different subgroups. The mixed model analysis was run for each variable separately in order to analyze the effect of the variable on the outcome as an outcome predictive factor. When significantly influencing the outcome, the variable was assessed as a confounder by fitting the mixed model analysis repeatedly in combination with the residual variables. Variables and outcomes of the statistical assessment were represented as mean with ± standard deviation (SD) when normally distributed. When data was not normally distributed as a median with range (minimum-maximum)(if not normally distributed). Statistical significance was assumed when p < 0.05.

Part of the quantitative meta-analysis was carried out using OpenMeta[Analyst] software (MetaAnalyst, Tufts Medical Center (Wallace *et al*., 2012)), which is the visual front-end for the R package (www.r-project.org; Metafor)^56^. A forest-plot was created to graphically display the estimated results from the included studies, along with the overall results In addition, OpenMeta[Analyst] was used to assess heterogeneity. Heterogeneity in a meta-analysis refers to the variation in outcomes between studies. To measure heterogeneity, the heterogeneity index (I_2_) was introduced. This measure can be interpreted as the proportion of total variability explained by heterogeneity and refers to the percentage of variation across studies^57^. I_2_ displays the inconsistency across studies and ranges from 0% (i.e., no heterogeneity) to 100% (i.e., the highest heterogeneity).

### Quality assessment

To assess the risk of bias of the results of the included studies, the researchers used two Cochrane tools. Version 2 of the Cochrane risk-of-bias tool for randomized trials (RoB 2)(*Version 22 August 2019*)^58^ was used for the randomized trials as this tool is the recommended tool to assess the risk of bias in such trials. The Risk Of Bias In Non-randomized Studies - of Interventions (ROBINS-I)(*Version 19 September 2016*)^59^ was used to assess the risk of bias in the results of non-randomized studies that compare health effects of two or more interventions. Both bias-assessment tools were filled in by two researchers separately (D.H. and E.K.). When no agreement could be achieved between the two reviewers on the quality of the trial, a third reviewer (R.B.) was consulted according to the Cochrane methodology^60,61^.

The quality of the evidence of the studies was graded according to the GRADE approach guidelines defined by Cochrane^62–71^. Two members of the team (D.H. and E.K.) independently reviewed each selected article for risk of bias using the Cochrane criteria checklist. When no agreement could be achieved between the two reviewers on the quality of the trial, a third reviewer (R.B.) was consulted according to the Cochrane methodology^60,61^. Assessing the risk of bias was performed by the criteria presented in Table 1, following standardized instructions which were published before^60,61,72–74^.

**Table 1:** Quality assessment of the individual papers.

Authors (ref)	Internal validity												Score	Quality
	1	2	3	4	5	6	7	8	9	10	11	12		
Meyerson, Lindblom *et al*.^45^	−	−	−	−	−	−	+	+	+	+	+	+	6	Moderate
Ebel, Rust *et al*.^10^	−	−	−	−	−	+	+	+	+	+	+	+	7	Moderate
Rainov, Fels *et al*.^24^	−	−	−	−	−	−	+	+	+	+	+	+	6	Moderate
Carroll, Joint *et al*.^9^	−	−	−	−	−	−	+	+	+	+	+	+	6	Moderate
Brown and Pilitsis^78^	−	−	−	−	−	+	+	+	+	+	+	+	7	Moderate
Rasche, Ruppolt *et al*.^47^	+	+	+	+	+	+	+	+	+	+	+	+	12	High
Hosomi, Saitoh *et al*.^13^	−	−	−	−	−	+	+	+	+	+	+	+	7	Moderate
Velasco, Arguelles *et al*.^76^	+	+	+	+	+	−	+	+	+	+	+	+	11	High
Pirotte, Voordecker *et al*.^79^	−	−	−	−	−	−	+	+	+	+	+	+	6	Moderate
Nguyen, Velasco *et al*.^77^	+	+	+	+	+	−	+	+	+	+	+	+	11	High
Anderson, Kiyofuji *et al*.^80^	−	−	−	−	−	−	+	+	+	+	+	+	6	Moderate
Perdok, van Dongen *et al*.^81^	−	−	−	−	−	−	+	+	+	+	+	+	6	Moderate
Lefaucheur, Drouot *et al*.^75^	+	+	+	+	+	+	+	+	+	+	+	+	12	High
Esfahani, Pisansky *et al*.^82^	−	−	−	−	−	−	+	+	+	+	+	+	6	Moderate
Raslan, Nasseri *et al*.^83^	−	−	−	−	−	+	+	+	+	+	+	+	7	Moderate
Tanei, Kajita *et al*.^42^	−	−	−	−	−	−	+	+	+	+	+	+	6	Moderate
Delavallee, Finet *et al*.^44^	−	−	−	−	−	+	+	+	+	+	+	+	7	Moderate
Buchanan, Darrow *et al*.^43^	−	−	−	−	−	+	+	+	+	+	+	+	7	Moderate
Sachs, Babu *et al*.^84^	−	−	−	−	−	+	+	+	+	+	+	+	7	Moderate
Slotty, Eisner *et al*.^85^	−	−	−	−	−	−	+	+	+	+	+	+	6	Moderate
Sokal, Harat *et al*.^40^	−	−	−	−	−	−	+	+	+	+	+	+	6	Moderate
Kolodziej, Hellwig *et al*.^55^	−	−	−	−	−	+	+	+	+	+	+	+	7	Moderate
Henssen, Kurt *et al*.^86^	−	−	−	−	−	−	+	+	+	+	+	+	7	Moderate

1. Was the method of randomization adequate?

2. Was the treatment allocation concealed?

3. Was the patient blinded to the intervention?

4. Was the care provider blinded to the intervention?

5. Was the outcome assessor blinded to the intervention?

6. Was the dropout rate described and acceptable?

7. Were all randomized participants analyzed in the group to which they were allocated?

8. Are reports of the study free of suggestion of selective outcome reporting?

9. Were the groups similar at baseline regarding the most important prognostic indicators?

10. Were co-interventions avoided or similar?

11. Was the compliance acceptable in all groups?

12. Was the timing of the outcome assessment similar in all groups?

(Questions derived from^60,61,72–74^).

+, criterion achieved; −, criterion not achieved; ∗, assessors initially disagreed

High [≥10/12]: Where criteria were not fulfilled, the conclusions of the study or review are thought very unlikely to have been altered.

Moderate [6–9/12]: Where criteria were not fulfilled, the conclusions of the study or review are thought unlikely to have altered the conclusions.

Low [6/12]: Where criteria were not fulfilled, the conclusions of the study or review are thought likely or very likely to alter had those criteria been fulfilled.

### Ethical approval

No ethical approval was needed for conducting this systematic literature review and meta-analysis.

### Consent for publication

The authors give consent for publication of the presented data.

## Results

**Figure 2:**
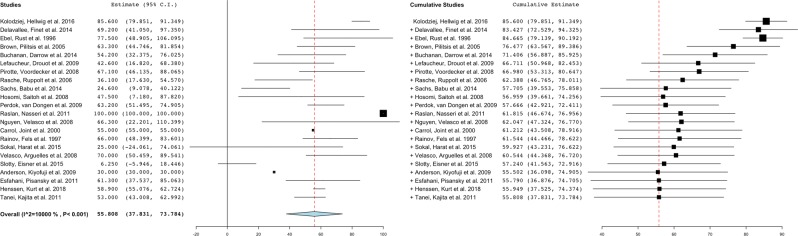
Forest-plot of pain relief (%) per study, the overall pain-relief (%) and heterogeneity as assessed by I_2_. Note: the study of Meyerson, Lindblom *et al*. 1993 could not be included in the forest-plot as this paper does not provide mean pain relief (%) data.

From the retrieved 287 articles, 23 papers could be included for data-extraction (Fig. 1). The included studies represented a total of 140 individual patients, 92 of which were women (65.7%) and 48 of which were men (34.3%). Subjects showed a mean age of 55.7 ± 13.3years. Median duration of pain prior to iMCS showed to be 6.4 years, ranging from 1.0–26.0 years. Mean preoperative and post-operative pain intensity scores showed to be 8.3 ± 1.5 and 3.9 ± 2.7, respectively. Median pain relief showed to be 64.8% (0–100%). A level 1 pain relief (pain reduction of 70–100%) was observed in 43.7% of the cases.A level 2 pain relief (pain reduction of 40–69%) was noted in 25.2% of the cases and a level 3 pain relief (pain reduction of 0–40%) was observed in 31.1% of the cases. Table 2 provides a detailed overview of the non-imputed data of the characteristics of the included papers. Figure 2 presents the created forest-plot of the mean pain relief achieved in each of the included studies and shows that heterogeneity was calculated to be 55.8% (95% confidence interval 37.8–73.8). Neurologic adverse events (n = 22) occurred in 11 patients or 22% of all complications, and included temporary, partial seizures in 9 patients (18%), temporary speech arrests in 1 patient (2%) and facial spasms in 1 patient (2%). Other complications were wound infections (25 patients; 12%), post-incision pain (1 patient (2%), epidural infection (1 patient; 2%) and post-operative trauma (1 patient; 2%).

**Table 2:** Characteristics from patients suffering from trigeminal neuropathic pain derived from the available eligible publications (non-imputed data).

	Ref.	Article type	N	Sex M/F	Mean age (years)	Diagnoses	Mean duration of pain (years)	Mean preoperative VAS	Mean VAS at last follow-up	**Mean period of follow-up (months)**	**Mean pain relief after iMCS (%)**	**Cases with significant pain control (%)**	**Complications (%)**	
													**Neurological**	**Other**
*1*	^55^	Retrospective analysis	9	1/8	57.1 ± 13.8	3;7;8;9;11	4.1 ± 3.4	—	—	—	85.6 ± 8.8	100	—	—
*2*	^44^	Observational study	7	5/2	52.6 ± 25.3	8;13	13.8 ± 8.5	8.5 ± 0.6	2.3 ± 3.3	121.2 ± 35.4	69.2 ± 38.0	83.3	28.6	42.9
*3*	^10^	Observational study	7	1/6	55.4 ± 15.3	1;7	10.6 ± 9.5	—	—	14.5 ± 0.0	77.5 ± 38.6	75.0	14.3	0.0
*4*	^78^	Observational study	9	5/4	59.3 ± 11.0	3;7;12;13	—	8.7 ± 1.8	3.4 ± 2.4	10.0 ± 0.0	63.3 ± 28.4	85.7	—	—
*5*	^43^	Observational study	5	1/4	58.2 ± 15.5	1	—	9.8 ± 0.5	3.5 ± 1.3	3.0 ± 0.0	54.2 ± 24.9	80	20.0	0.0
*6*	^75^	RCT	7	1/6	58.9 ± 16.6	4;6;7;9;12;15	11.4 ± 15.5	9.5 ± 0.7	—	—	42.6 ± 34.8	60.0	0.0	14.3
*7*	^79^	Observational study	7	2/5	52.1 ± 12.4	4;11;13	—	7.7 ± 0.5	2.6 ± 2.3	—	67.1 ± 28.3	85.7	0.0	14.3
*8*	^47^	Retrospective analysis	10	0/10	64.1 ± 10.1	1;4;9;12	6.3 ± 3.3	8.0 ± 0.0	5.5 ± 3.0	4.2 ± 3.5	36.1 ± 29.8	50	—	—
*9*	^84^	Retrospective analysis	8	4/4	48.4 ± 7.4	9;13	—	7.2 ± 1.2	5.4 ± 1.7	13.1 ± 15.0	24.6 ± 22.4	12.5	37.5	12.5
*10*	^13^	Observational study	5	3/2	54.6 ± 17.8	3;11;13	3.5 ± 0.7	—	—	54 ± 5.7	47.5 ± 46.0	50	—	—
*11*	^81^	Retrospective analysis	7	3/4	53.3 ± 7.4	3;9;10;11;13	—	8.1 ± 1.6	3.0 ± 1.7	21.6 ± 3.3	63.2 ± 15.8	100	—	—
*12*	^83^	Retrospective analysis	11	3/8	47.4 ± 13.0	1;4;9;10;11;12	4.7 ± 3.3	—	-	31.6 ± 21.2	100 ± 0.0*	100	—	—
*13*	^77^	Double-blinded crossover trial	4	2/2	53.8 ± 18.1	11;13	3.75 ± 2.2	—	—	—	66.3 ± 45.0	75	—	—
*14*	^9^	Observational study	3	1/2	71.3 ± 15.0	3;10;11	6.7 ± 4.0	—	—	31.0*	55.0*	100	0.0	33.3
*15*	^82^	Observational study	3	2/1	61.3 ± 21.0	7;14	9.3 ± 1.2	9.0*	0.0*	—	87.7 ± 11.0	100	66.7	33.3
*16*	^86^	Observational study	14	6/8	58.9 ± 7.3	2;4;9;10;11;12	8.7 ± 6.5	8.9 ± 1.1	10.2 ± 18.9	36.0 ± 0	40 ± 28.9	42.9	7.1	7.1
*17*	^42^	Retrospective analysis	5	3/2	53.0 ± 11.4	11	—	—	—	—	75.0 ± 10.0	100.0	20	0.0
*18*	^24^	Case report	2	0/2	51.5 ± 12.0	5;12	15.0*	7.5 ± 0.7	2.5 ± 0.7	44.5 ± 46.0	66.0 ± 12.7	100.0	50.0%	0.0
*19*	^40^	Retrospective analysis	2	0/2	61.0 ± 4.2	2	7.0 ± 1.4	8.5 ± 0.7	6.5 ± 3.5	182.0 ± 110.3	25.0 ± 35.4	50.0	0.0	0.0
*20*	^45^	Observational study	10	3/7	51.2 ± 8.6	10;11;13	6.3 ± 3.9	—	—	—		0.0*	0.0	0.0
*21*	^76^	Observational study	2	1/1	47.5 ± 6.4	7;11	3.9 ± 4.4	10.0 ± 0.0	3.0 ± 1.4	—	70.0 ± 14.1	100.0	—	—
*22*	^85^	Retrospective analysis	2	1/1	70.5 ± 12.0	14	6.9 ± 0.1	9.0 ± 0.0	8.5 ± 0.7	25.0 ± 4.2	6.25 ± 8.8	100.0	—	—
*23*	^80^	Case report	1	0/1	54.0	13	14.0	10.0	7.0	5.0	30.0	100.0	0.0	0.0

1 = Anesthesia dolorosa; 2 = Atypical facial pain; 3 = Brainstem lesion; 4 = Dental avulsion pain; 5 = Glossopharyngeal neuralgia; 6 = Neurofibromatosis type 1; 7 = Post-herpetic neuropathic pain; 8 = Post-neurosurgical pain; 9 = Post-surgical pain; 10 = Post-traumatic pain; 11 = Symptomatic trigeminal neuralgia; 12 = Trigeminal neuralgia; 13 = Trigeminal neuropathic pain; 14 = Trigeminal deafferentation pain; *= limited subjects in subanalysis due to missing information; VAS = Visual analogue scale; − = Missing.

CNOP was found to be caused by various diagnoses, which are summarized in Table 3. Using an anatomical-functional classification system, these diagnoses were categorized as: Group 1) Absence of intrinsic trigeminal nerve lesions (including trigeminal neuralgia; n = 14); Group 2) Trigeminal nerve lesions (including anesthesia dolorosa, dental avulsion pain, neurofibromatosis type 1, post-herpetic neuropathic pain, post-surgical pain, post-traumatic pain, trigeminal neuropathic pain and trigeminal deafferentation pain; n = 79); Group 3) (Supra)nuclear lesions (including pain after brain(stem) lesions (post-stroke pain), post-neurosurgical pain, symptomatic trigeminal neuralgia; n = 36); and Group 4) Dysfunctional pain syndromes (including atypical facial pain, idiopathic facial pain; n = 11).

**Table 3:** Overview of diagnoses causing orofacial pain and efficacy of iMCS.

Causes of orofacial pain	Anatomical-functional classification	Number of patients described	Number of patients with missing data	Percentage of all patients	Mean pain reduction (%)based on the pre- and post-operative VAS	Standard deviation (±SD)	Median pain reduction (%)based on the pre- and post-operative VAS	Range
Anesthesia dolorosa	Group 2	11	4	7.9%	73.7%	±28.6%	80.0%	20–100%
Atypical facial pain	Group 4	4	0	2.9%	23.8%	±27.5%	22.5%	0–50%
Brainstem lesions	Group 3	9	3	6.4%	62.8%	±28.0%	65.0%	15–100%
Dental avulsion pain	Group 2	8	3	5.7%	34.4%	±23.7%	39.0%	11–69%
Neuro-fibromatosis type 1	Group 2	1	1	0.7%	5.0%	N/A	5.0%	N/A
Post-herpetic neuropathic pain	Group 2	8	2	5.7%	86.7%	±45.6%	85.0%	70–100%
Post-neurosurgical pain	Group 3	4	1	2.9%	63.7%	±45.6%	90.0%	11–90%
Post-surgical pain	Group 2	11	3	7.9%	34.5%	±34.0%	22.5%	0–90%
Post-traumatic pain	Group 2	6	2	4.2%	53.8%	±36.8%	52.5%	10–100%
Symptomatic trigeminal neuralgia (post-stroke, MS-lesions)	Group 3	23	8	16.4%	69.3%	±21.7%	70.0%	20–100%
Trigeminal neuralgia	Group 1	14	2	10.0%	60.2%	±28.5%	66.5%	0–100%
Trigeminal neuropathic pain	Group 2	30	6	21.4%	55.4%	±35.0%	70.50%	0–100%
Trigeminal deafferentation pain	Group 2	4	0	2.9%	43.9%	±43.8%	45.8%	0–84%
Idiopathic facial pain	Group 4	7	2	5%	45.2%	±18.9%	50.0%	15–67%

Group 1 = Absence of intrinsic trigeminal nerve lesion; Group 2 = Trigeminal nerve lesion; Group 3 = Nuclear- and supranuclear lesion; Group 4 = Dysfunctional pain syndrome; N/A = Not applicable; VAS = Visual analogue scale.

### Linear mixed-model analysis

Linear mixed-model analysis showed a mean pain relief of iMCS of 67.4% ± 8.7% in Group 1, 53.3 ± 4.5% in Group 2, 66.0 ± 5.9% in Group 3 and 40.8 ± 9.2%% in Group 4. Pairwise comparisons showed a statistically significant difference in pain relief between patients belonging to Group 1 and Group 4, in favor of patients within Group 1 (p = 0.030; 95%-CI = 2.7–50.7). Also, statistically significant differences in pain relief were found between Group 2 and Group 3 (p = 0.049; 95%-CI = −25.4-0.05, in favor of Group 3) and between Group 3 and Group 4 (p = 0.017; 95%-CI = 4.8-45.6, in favor of Group 3). The duration of pain prior to iMCS did not contribute to the outcome (p = 0.33), nor did the preoperative pain intensity (p = 0.88). Neither sex of the patient and the preoperative pain intensity score were found to be a confounder (p = 0.07; p = 0.13; p = 0.39).

### Quality assessment

Risk of bias as assessed by the RoB2 showed that all included papers had a low overall risk-of-bias judgement^47,75–77^. Risk of bias as assessed by the ROBINS-I showed that all included papers had a moderate overall risk-of-bias judgement^9,10,13,24,40,42–45,55,78–86^. Table 1 provides an overview of the quality of the evidence of the included papers, respectively. This overview shows that the evidence of the four papers were of high quality^47,75–77^. The other papers were assessed as being of low/moderate quality of evidence^9,10,13,24,40,42–45,55,78–86^.

## Discussion

This study shows that a broad variety of diagnoses, which all can cause CNOP, have been described in the iMCS literature. This broad variety of disorders causes a relatively heterogeneous group of patients, possibly explaining the reported variable outcome of iMCS. This study furthermore suggests that the effectiveness of iMCS is associated with the neuroanatomic location of the lesion causing CNOP. It was found that the outcomes of iMCS were most optimal in patients in whom the integrity of the trigeminal nerve was not affected. More specific, patients suffering from CNOP caused by (supra)nuclear lesions respond more favorable than others.

### Effectiveness of iMCS and confounding factors

iMCS is considered a last-resort, experimental technique for patients with CNOP who do not respond to regular treatments. However, there is no international consensus on how to define a patient as a non-responder during treatment of CNOP. Nevertheless, all patients in the included papers were described as sufferers from severe CNOP that did not respond to regular treatments (i.e. oral analgetics, including opioids and anti-epileptic medication). In addition, most patients in the included papers were also reported to be non-responders less regular treatments (i.e. cervical spinal cord stimulation, thalamic deep brain stimulation).

With regard to the effectiveness of iMCS, the neuromodulation society is divided into believers and non-believers^49^. The present study shows that in 68.9% of the cases, a clinically significant pain reduction was achieved. A narrative review reported that of the 100 reviewed patients suffering from CNOP, 84 experienced good pain control by iMCS^48^. These authors also acknowledged that CNOP can be caused by a large variety of disorders via different pathophysiological pathways, although they did not discuss the possible confounding effects of this variety^48^.

Other confounding factors, next to the variety of diagnoses, have been investigated as well. For example, an intact corticospinal tract has been considered mandatory for adequate analgesia^14^, whereas others found that neither age, sex, preoperative motor status, pain characteristics, etiology of pain, quantitative sensory testing or neurophysiological monitoring were of significant influence. Only the pain intensity scores in the first months of follow-up seemed to be of significant influence. The influence of the duration of pain before surgery and the outcome of iMCS was found significant in one study, whereas other studies did not find this to be significant^13,21,87^. Also, the use of several transcranial magnetic stimulation (TMS) protocols has been demonstrated to be predictive of iMCS outcome^13,88–90^. For example, the study of André-Obadia showed in 2006 that high-frequency rTMS may become useful to select candidates for iMCS. Nevertheless, all papers discuss that placebo effects are potentially powerful and should be controlled for, if possible. A previous paper of our group also investigated outcome prediction factors involved in iMCS outcome. These factors included the sex of the patient, the origin of the lesion within the somatosensory nervous system, the preoperative VAS score, the preoperative use of rTMS, the preoperative intake of opioids and the duration of the follow-up period^50^.However, with the exception of the possible effect of the localization of the lesion, these factors were not reproduced in the present research.

### Mechanisms of action of iMCS

Every thalamic nucleus is known to receive feedback from the sixth layer of the motor cortex^91^. That these connections and the zona incerta are involved in the regulation of pain, via the GABA-ergic pathways, has been shown before^92–94^. The opioidergic system has also been described to be involved in pain relief by iMCS as it is thought to modulate the descending volleys towards the PAG and related nuclei^95–99^. Activation of the striatal dopaminergic system, on the other hand, seems to be involved as well^100,101^. The release of noradrenalin (locus coeruleus)^102^ and serotonin (the rostroventromedial medulla)^103,104^ has been described to be involved in the analgesic effects of iMCS as well. The role of N-meyhyl-D-aspartate receptors in explaining the effects of iMCS has also been reported as important^105,106^. Finally, mechanisms of the descending volleys in the spinal cord have also been described^103,104,107^. The activation of stellate interneurons in the fourth layer of the cerebral cortex is assumed to explain such wide-spread effects of iMCS^108–114^.

Next to these neuroanatomical substrates, corollary discharges have recently been investigated to explain the effect of iMCS. A corollary discharge is a copy of a motor command that is sent to the muscles to produce a movement. This corollary is directed to other brain regions to inform them of the impending movement but does not inflict movement on its own^115^. It has been discussed that sensory feedback comes from the peripheral nerves, the visual input, but also from the motor cortex itself. Therefore, a possible mechanism of action of iMCS is thought to lie in corollary discharges from the primary motor cortex that counterbalance other feedback deficiencies^115^. Although several clinical trials showed a significant difference in analgesic effects between sham and active stimulation of the primary motor cortex^76,116,117^, the placebo effect is known to play a role in pain relief^118^.

### Strengths and limitations

One of the strengths of this study concerns its novel attempt to integrate unique data sources to investigate an understudied research area. Nevertheless, as the quality of the retrieved literature was considered to be moderate/low, the conclusions must be interpreted with caution. The inconsistent use of the nomenclature of several diagnoses forms another limitation of this study as it complicates the analysis of groups of diagnoses. In addition, the lack of psychometric properties of the VAS scores that were used as an outcome measurement in all the included studies forms another limitation of these studies and the present meta-analysis as it hampers the direct translation of these results to clinical decision making. For example, the included papers often did not report on modifications in quality of life scores before and after iMCS. The absence of a large randomized controlled trial with regard to iMCS CNOP forms an important limitation of this meta-analysis. The absence of such well-designed trials indicate a crucial shortage in the scientific literature with regard to iMCS and CNOP. Furthermore, part of the scientific literature could not be included in this analysis due to the fact that these papers did not meet the strict, predefined inclusion criteria^20,41,119,120^. It is known that randomized controlled trials are well-suited to investigate the influence of the placebo response and to evaluate the true treatment effect in an appropriate manner. The relative absence of such well-designed trials indicate a crucial shortage in the scientific literature with regard to iMCS and CNOP. Based on other invasive treatment studies, the placebo-effect is possibly stronger as compared to studies in which less invasive treatments were carried out. Therefore, it is not possible to rule out or determine the placebo-effect in the included studies or the current paper. This limitation provokes a risk of bias that precludes the drawing of a sound conclusion. Finally, it is for ethical reasons impossible to perform a sham operation to provide a control group. Possibly a double blinded on/off-phase trial could be a valuable addition with regard to the lack of a control group.

## Conclusions

The overall analgesic effect of iMCS might be relevant for CNOP patients who do not respond to other treatments. The best results of iMCS are achieved in patients with CNOP etiologies affecting the central portion of the trigeminal system. No other factors were found to significantly influence the outcome of iMCS in CNOP disorders. However, due to the small sample size, the relatively poor quality of the analyzed literature and the inconsistent use of diagnoses, this statement needs further exploration in future studies.

## Data Availability

Data and material can be requested by contacting the corresponding author.
